# Reassessing HIV Detection Strategies: An Analysis of Opportunistic Screening vs. Indicator-Condition-Driven Diagnosis in Valencia, Spain

**DOI:** 10.1007/s10900-024-01326-9

**Published:** 2024-02-26

**Authors:** Enrique Ortega, María Dolores Ocete, María Martínez-Roma, Concepción Gimeno, Neus Gómez, Moisés Diago, Alba Carrodeguas, Diogo Medina, Miguel García-Deltoro

**Affiliations:** 1 Fundació Investigació Hospital General Universitari de Valencia, Valencia, Spain; 2 Consorci Hospital General Universitari de Valencia, Valencia, Spain; 3 Facultad de Medicina, Universidad de Valencia, Valencia, Spain; 4 Gilead Sciences, Madrid, Spain

**Keywords:** HIV, Screening, Diagnosis Strategy, Linkage to care

## Abstract

Our study assessed the characteristics of people living with HIV (PLWH) detected via opportunistic screening in Valencia (Spain) to determine diagnoses potentially missed under a more restrictive, indicator-condition diagnostic strategy. We conducted a retrospective analysis of electronic health records of 97 PLWH diagnosed between April 2019 and August 2022. The main outcomes reported were patient CD4^+^ T cell count, known HIV risk factors at diagnosis, and missed opportunities for diagnosis, defined as the failure of a previously untested patient to undergo HIV testing despite attending previous visits to healthcare facilities prior to diagnosis. Successful linkage to care was achieved for 95.9% of diagnosed patients. Half of the PLWH were diagnosed late, while 47.8% did not meet the criteria for indicator-condition-driven HIV diagnosis at the time of their diagnosis. Additionally, 52.2% did not receive HIV testing despite an average of 5.1 ± 6.0 healthcare visits in the 12 months prior to diagnosis. Spaniards had more missed opportunities for diagnosis than foreigners (64% vs. 40%, *p* = 0.02). Depending solely on an indicator-condition-driven HIV diagnosis approach could result in 47.8% of cases being missed. Including “migrants” as a testing criterion could lower missed diagnoses to 25.3% but might create inequities in prevention access. In conclusion, our findings provide valuable insights to enhance HIV testing, early diagnosis, and linkage to care. While it is crucial to uphold the indicator-condition-driven HIV diagnosis as baseline practice, improving screening strategies will decrease late diagnoses and missed opportunities, thereby effectively contributing to end the epidemic.

## Introduction

The Joint United Nations Programme on HIV-AIDS (UNAIDS) urges countries to upscale human immunodeficiency virus (HIV) testing to ensure that 95% of people living with HIV (PLWH) are aware of their status [1, 2]. However, late HIV diagnosis remains a significant challenge in several Western European countries, including Spain. In 2021, 52.5% of patients in Western Europe and 49.8% of patients in Spain were diagnosed late (defined as having a CD4^+^ T cell count < 350 cells/μL), highlighting the need to improve HIV screening strategies toward early diagnosis [3, 4]. 

Previously, we reported results from a large-scale opportunistic screening and linkage to care project across various departments within the Consortium General University Hospital of Valencia (Consorcio Hospital General Universitario de València, CHGUV), including 26 primary care centers, 6 sexual and reproductive health centers, 3 mental health centers, 3 addiction treatment centers, selected hospital departments, outpatient hospital clinics, and a penitentiary facility (manuscript submitted for publication). From February 2019 to March 2020, this project identified a 0.11% undiagnosed HIV prevalence in 13,061 patients aged between 16 and 80 – over 5 times greater than the undiagnosed HIV infection prevalence of 0.02% estimated for Spain in 2021 [5, 6]. Though cost-effectiveness thresholds for Spain are not established, HIV screening has been proven to be cost-effective in the US with undiagnosed HIV prevalence as low as 0.05–0.1%, in the UK with ≥ 0.2% prevalence, and in Portugal with ≥ 0.05% prevalence [7–9].

There is a robust ongoing debate regarding which HIV screening strategy is the most effective for achieving UNAIDS goals in Europe, with the terms “testing”, “screening”, and “diagnosis” mistakenly being used interchangeably. “Testing” refers to the mere technical process of using immunoassays or molecular assays to identify infection; “screening” refers to large-scale testing programs intended to identify infections in apparently healthy individuals; while “diagnosis” pertains to the process of identifying infections based on their signs and symptoms [10]. Further understanding of differences between screening strategies is equally crucial. Universal, mass, organized, or population-based HIV screening targets entire populations or major demographic subgroups (e.g., age groups). While closely related in purpose, opportunistic and case-finding HIV screening projects are limited to those seeking care, integrating serologies with other blood tests during clinical encounters held for other reasons for increased efficiency. Lastly, targeted, selective, or high-risk HIV screening programs limit eligibility to individuals with characteristics associated with an increased risk, such as belonging to key groups: men who have sex with men (MSM), transgender individuals, sex workers, people who inject drugs (PWID), people in prisons, migrants, and people experiencing homelessness. While often labeled as screening, testing based on signs and symptoms of indicator conditions is inconsistent with the concept of screening and should be considered diagnostic [10]. 

Public health authorities hold varying opinions on the best approach to HIV screening. The World Health Organization (WHO) recommends targeted HIV screening of key populations in all clinical settings [11]. In 2006, the US Centers for Disease Control and Prevention (CDC) recommended universal HIV screening of patients aged 13 to 64 years in areas where undiagnosed HIV prevalence is 0.1% or higher [12]. Meanwhile, in 2018, the European Centre for Disease Prevention and Control (ECDC) proposed targeted HIV screening of key populations, reserving universal HIV screening for areas where the prevalence of undiagnosed HIV infection is 1.0% or higher [13]. 

In 2014, the Spanish Ministry of Health advised screening sexually active individuals aged 20 to 59 years who visit primary care facilities, require a blood draw for any reason, and reside in Spanish provinces with an HIV incidence above the 75th percentile in the last 3 years [14]. Since 2020, the Spanish Society of Emergency Medicine (SEMES) has recommended indicator-condition-driven HIV diagnosis in patients presenting with sexually transmitted infections (STIs), herpes zoster, and community-acquired pneumonia, and in patients reporting recent risk exposures, such as chemsex and a recent need for post-exposure prophylaxis (PEP) [15]. Although early reports show the strategy has been effective, it may be insufficient as a standalone strategy to end the HIV epidemic, as up to 60% of PLWH are never symptomatic before progressing to the acquired immunodeficiency syndrome stage [16, 17]. 

To date, testing strategies implemented on the ground in Europe have primarily been risk-based and have not been effective in identifying all infected individuals [13]. Failure to implement screening strategies often results in missed opportunities for detection, given the significant proportion of PLWH seeking health services prior to diagnosis [18–22]. Opportunities to halt transmission are also lost, with 3.38 to 4.14 secondary infections expected for every person not diagnosed and linked to care [5]. 

Our study aimed to describe patient characteristics of PLWH diagnosed by way of an opportunistic screening program in a health consortium in Valencia, Spain, and to estimate the proportion of PLWH that would have been diagnosed had a more restrictive, indicator-condition diagnostic approach been in place instead.

## Methods

### Project Design

We conducted a retrospective analysis of the electronic health records of PLWH diagnosed at CHGUV between April 2019 and August 2022. HIV testing in the emergency department (ED) was performed in patients fulfilling any of the SEMES criteria [15], while the remaining tests were performed through opportunistic screening in other settings: primary care, infectious diseases and other departments within our healthcare consortium. Participation in the opportunistic screening was offered to all patients ≥ 18 years old. Tests were performed in individuals who signed the informed consent.

### Main Outcomes and Measures

Variables considered were sex, age at diagnosis, country of origin, year of diagnosis, and clinical setting of visit. The main outcomes reported were patient CD4^+^ cell count, known HIV risk factors and exposures at diagnosis, and missed opportunities for diagnosis, defined as the failure of a previously untested patient to obtain HIV testing despite attending 1 or more visits in healthcare facilities in the 12 months prior to diagnosis.

### Statistical Analysis

All data points were analyzed both descriptively and inferentially. The descriptive statistics are reported as percentages or mean ± standard deviation (SD). A Chi-square test was used to assess the associations between categorical variables. The results from the Chi-square test have been presented wherever applicable.

## Results

We examined the records of a total of 97 patients diagnosed with HIV infection at CHGUV between April 2019 and August 2022. The demographic characteristics of these patients are shown in Table 1. Overall, 95.9% (*n* = 93) were successfully linked to care post-diagnosis. We had data on the country of origin for 98% (*n* = 95) of PLWH: 53.7% (*n* = 51) were Spaniards; 24.2% (*n* = 23) hailed from Latin America and the Caribbean; 10.5% (*n* = 10) were from Africa; 10.5% (*n* = 10) were from other European countries; and 1.1% (*n* = 1) were from Asia. PLWH of non-Spanish nationalities came from 23 different countries, of which the largest groups were from Colombia (13.6%, *n* = 6), Romania (11.4%, *n* = 5), Venezuela (11.4%, *n* = 5), and Equatorial Guinea (9.1%, *n* = 4).

**Table 1:** Demographics and clinical characteristics of patients diagnosed with HIV at CHGUV between April 2019 and August 2022

**People diagnosed with HIV, ** ***n*** ** (%)**	*N* = 97 (100%)
April – December 2019	11 (11.3%)
January – December 2020	9 (9.3%)
January – December 2021	41 (42,3%)
January – August 2022	36 (37.1%)
**Sex (male)**, ***n*(%)**	84 (86.6%)
**Age, mean ± SD [Q1-Q3]**	39.7 ± 12.6 [16–74]
**Country of origin**, ***n*(%)**	
Spaniards	51 (52.6%)
Latin America and the Caribbean	23 (23.7%)
Africa	10 (10.3%)
Other European countries	10 (10.3%)
Asia	1 (1.0%)
Not known	2 (2.1%)
**CD4 + T cell count (cells/μL)**, ***n*(%)**	
≥ 500 (early presentation)	23 (25.0%)
351–499 (early presentation)	23 (25.0%)
201–350 (late presentation)	20 (21.7%)
≤ 200 (advanced HIV disease)	26 (28.3%)
**Linked to care**, ***n*(%)**	93 (95.9%)

**Fig. 1:**
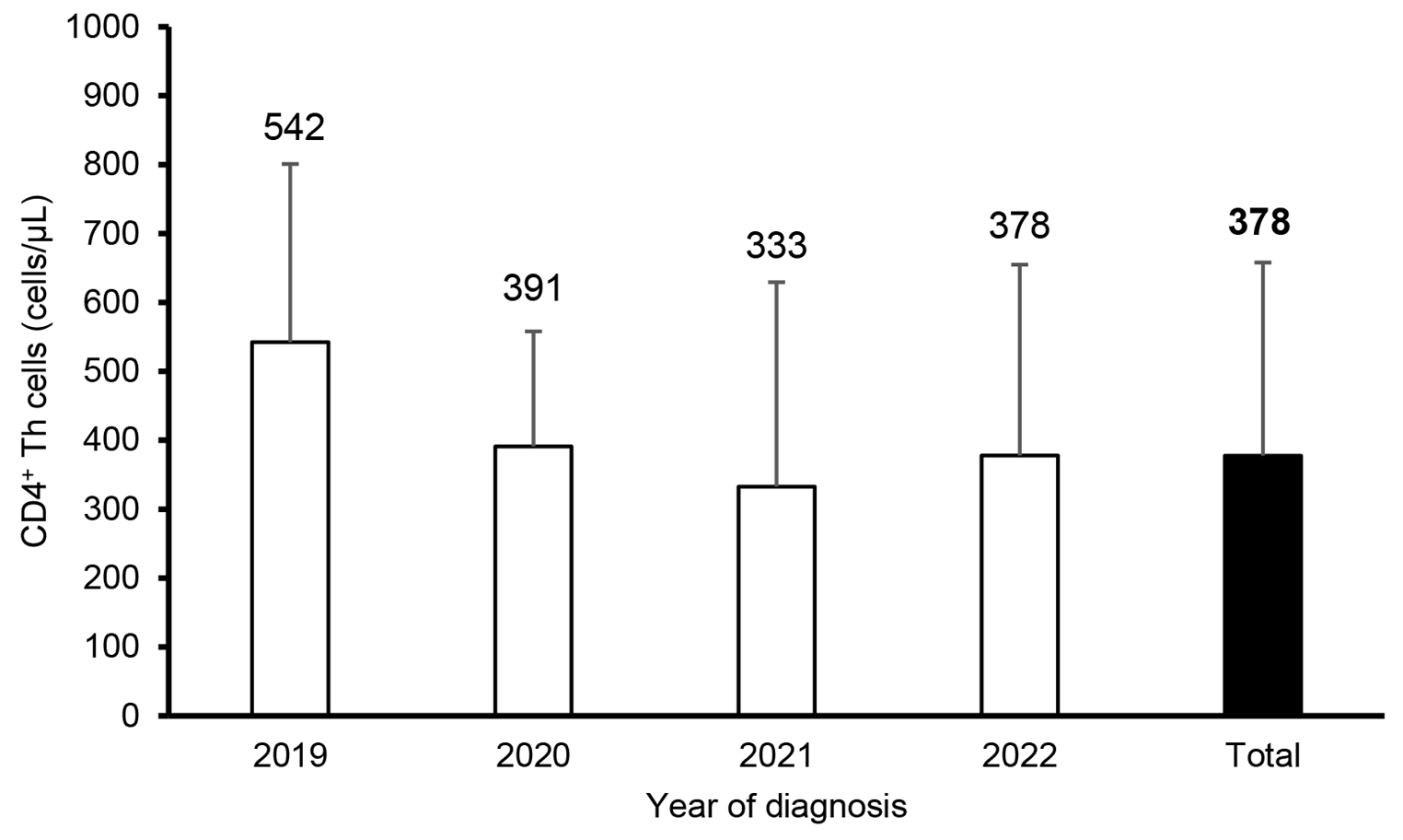
Newly diagnosed PLWH CD4 + T cell count distribution by year of diagnosis, average and standard deviation. *N* = 97 (2019 *n* = 11; 2020 *n* = 9; 2021 *n* = 41; 2022 *n* = 36)

Information on the clinical setting of the visit was available for 93% (*n* = 90) of the PLWH. Of these, 42.2% (*n* = 38) were diagnosed in primary care centers, 16.7% (*n* = 15) in the infectious diseases department, 13.3% (*n* = 12) in the ED, and 27.8% (*n* = 25) in over 10 other settings, including dermatology, gastroenterology, gynecology, hematology, internal medicine, neurology, pneumology, surgery and urology units, and prison. CD4^+^ T cell count information at diagnosis was available for 95% (*n* = 92) of the PLWH, with an average count of 382 ± 280 cells/μL. Half of the PLWH presented at late stages of the infection. Table 1 shows the distribution of PLWH by stage of HIV presentation at diagnosis, and Fig. 1 shows the distribution of CD4^+^ T cell counts over time.

Additionally, 47.8% (*n* = 43) of PLWH did not meet any of the SEMES criteria for HIV testing at the time of diagnosis (Table 2), while 42.2% (*n* = 38) had a prior STI, 7.8% (*n* = 7) had herpes zoster, 4.4% (*n* = 4) had pneumonia, 3.3% (*n* = 3) reported a history of chemsex use, and only 1.1% (*n* = 1) had a history of PEP use. Percentages do not add up to 100% as 6.7% (*n* = 6) of PLWH had overlapping eligibility criteria. A CD4^+^ T cell count is available for 95% (*n* = 41) of the patients who did not meet the SEMES criteria, of whom 21 (51.2%) had CD4 ≤ 350, with an average of 192 ± 112 cells/μL. Table 2 displays detailed data on observance of SEMES indicator-condition-driven HIV diagnosis criteria. We found no statistically significant differences in the proportion of PLWH presenting with SEMES criteria for HIV testing or at a late stage of infection according to sex, country of origin, and setting of diagnostic visit.

**Table 2:** Newly diagnosed PLWH observance of SEMES indicator-condition-driven HIV diagnosis criteria per diagnosing clinical setting

Observed indicator conditions	Emergency department, n (%)	Primary care, n (%)	Infectious diseases, n (%)	Other departments, n (%)	Total, n (%)
**None**	**3 (25.0%)**	**18 (47.4%)**	**10 (66.7%)**	**12 (48.0%)**	**43 (47.8%)**
**One or more**	**9 (75.0%)**	**20 (52.6%)**	**5 (33.3%)**	**13 (52.0%)**	**47 (52.2%)**
Prior STI	9 (75.0%)	20 (52.6%)	3 (20.0%)	6 (24.0%)	38 (42.2%)
Herpes zoster	0 (0.0%)	1 (2.6%)	1 (6.7%)	5 (20.0%)	7 (7.8%)
Pneumonia	1 (8.3%)	0 (0.0%)	2 (13.3%)	1 (4.0%)	4 (4.4%)
Chemsex	1 (8.3%)	0 (0.0%)	0 (0.0%)	2 (8.0%)	3 (3.3%)
PEP	1 (8.3%)	0 (0.0%)	0 (0.0%)	0 (0.0%)	1 (1.1%)
Mononucleosis syndrome	0 (0.0%)	0 (0.0%)	0 (0.0%)	0 (0.0%)	0 (0.0%)
**Total PLWH**	**12 (13.3%)**	**38 (42.2%)**	**15 (16.7%)**	**25 (27.8%)**	**90 (100.0%)**

PEP: post-exposure prophylaxis; STI: sexually transmitted infection. NB: Column percentages do not add up to 100% as 6.7% (*n* = 6) of PLWH had overlapping eligibility criteria

**Fig. 2:**
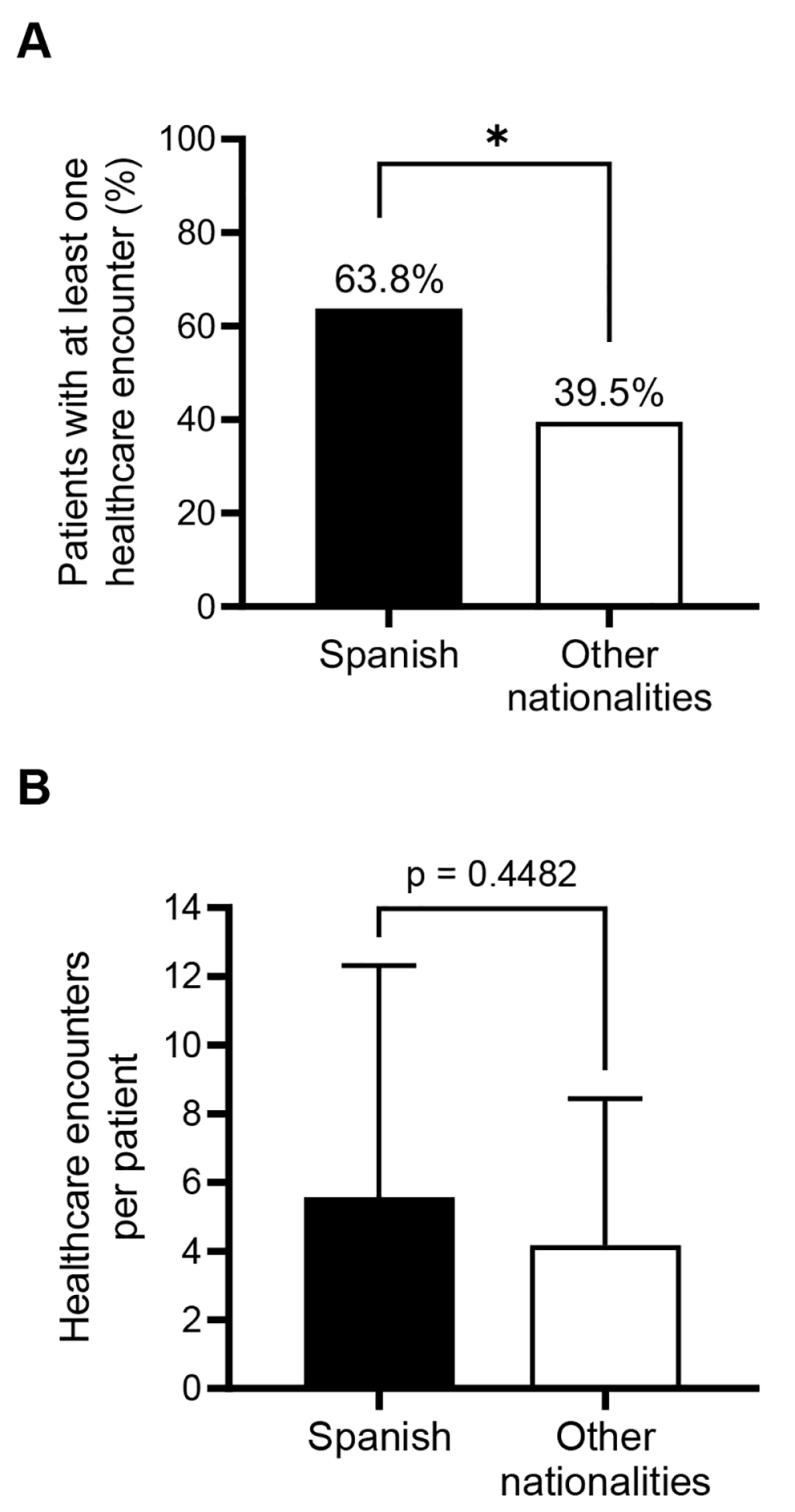
Missed opportunities for diagnosis in the previous year according to nationality. *N* = 97 (Spanish *n* = 51; other nationalities *n* = 46). (**A**) Percentage of patients with at least 1 health encounter during the year prior to diagnosis. *: *p* < 0.05. (**B**) Mean number of healthcare encounters per patient (average and standard deviation)

Information on missed opportunities for diagnosis was available for 93% (*n* = 90) of PLWH. Of these, 52.2% (*n* = 47) failed to receive HIV testing despite attending an average of 5.1 ± 6.0 (1–29) healthcare visits in the 12 months prior to diagnosis. We found statistically significant differences in the proportion of PLWH with missed opportunities for diagnosis according to country of origin. Missed opportunities affected 63.8% of Spaniards and 39.5% of PLWH of other nationalities, χ² (1, *n* = 90) = 5.31, *p* = 0.02 (Fig. 2A). Spanish patients attended 5.6 ± 6.8 healthcare visits during the previous year compared to 4.1 ± 4.3 in case of PLWH from other nationalities (Fig. 2B), but the difference was not statistically significant (*p* = 0.45).

## Discussion

This study provides a comprehensive examination of the demographics, clinical characteristics, and testing patterns of patients diagnosed with HIV at CHGUV from April 2019 to August 2022. The findings shed light on the epidemiology of HIV in our setting and highlight key areas for intervention to improve early diagnosis and linkage to care. The decrease in PLWH diagnosed in 2020 is consistent with the known reduction in testing activity of all types during the COVID-19 pandemic lockdown periods that year. However, it is important to clarify that variations in the number of cases reported per year should not be conflated with prevalence since the total number of patients tested has not been reported.

Our data indicate a predominance of HIV diagnoses among males (86.6%), consistent with epidemiological trends observed in Spain (86.1%) in the same period [4]. The mean age at diagnosis was approximately 40 years, suggesting that HIV continues to affect individuals in their most productive years, underscoring the public health implications of this infection. Most PLWH (95.9%) were linked to care post-diagnosis, reflecting the effectiveness of our current referral and linkage systems. We call upon national and autonomous community HIV reporting to start including data on the number of PLWH linked to care post-diagnosis, given its importance to achieving elimination goals.

Nonetheless, the proportion of PLWH diagnosed at late stages of infection (50.0%) remains a concern. Late-stage diagnosis not only worsens prognosis but also increases the likelihood of onward transmission, reinforcing the need for early diagnosis strategies. This is of concern, especially given the apparent downward trend in average CD4^+^ T cell count over time as shown in Fig. 1, suggesting a worsening trend of late HIV presentation in the population.

Our data indicate that HIV diagnoses among foreigners are more prevalent in Valencia (46.3%) than the reported figures for Spain (38.6%)^4^. The diversity in the country of origin of PLWH reflects global migration trends and underscores the importance of culturally sensitive HIV testing and prevention interventions. The sizable proportions of PLWH originating from Latin America and the Caribbean (24.2%), Africa (10.5%), and other European countries (10.5%) suggest that these populations may require enhanced intervention. Including “migrants” as an additional criterion for HIV testing, along with indicator conditions, would lower the proportion of PLWH with missed opportunities for diagnosis to 25.3%, while requiring only minimal additional testing. However this could potentially create inequity in prevention access, as asymptomatic Spaniards would likely continue to face a higher likelihood of missed diagnostic opportunities.

The diagnostic setting distribution shows a substantial number of PLWH diagnosed in primary care (42.2%) and a variety of other clinical settings. This highlights the essential role of non-HIV specialists in early detection and the importance of incorporating routine HIV screening in different clinical settings. The results in the ED, which did not participate in the screening, show that the SEMES indicator-condition-driven HIV diagnosis criteria were correctly applied in this setting, since 75.0% of PLWH diagnosed met one or more of the criteria. The high adherence to SEMES criteria in the ED suggests a strong adoption of the diagnostic strategy among providers. However, it does not highlight potential gaps in detecting undiagnosed PLWH who present to the ED without indicator conditions and thus miss out on HIV testing. In fact, only 13.3% of PLWH were diagnosed in the ED, while the remaining 86.7% were detected in the departments participating in the screening. In our opinion, this reinforces the need to also implement opportunistic screenings in ED, given the high diagnostic potential of this setting [23–27]. A substantial proportion of PLWH (51.3%) diagnosed in other healthcare settings did not meet any of the SEMES criteria. This underscores the limitations of indicator-condition-driven diagnostic strategies in tackling the epidemic. According to our dataset, relying solely on SEMES criteria would have led to missed diagnostic opportunities in 47.8% of PLWH.

Indeed, our results reveal missed opportunities for earlier diagnosis, with over half (52.2%) of the patients having had multiple healthcare contacts (x̄5.1 ± 6.0) in the year preceding diagnosis without being tested for HIV. Our data show higher proportions of patients with missed opportunities for diagnosis than in a similar series in Portugal^22^ (52.2% vs. 36.1%), and higher number of previous healthcare contacts (x̄5.1 ± 6.0 vs. x̄1.4 ± 2.9). Significant differences were observed in our case in missed opportunities for diagnosis according to the country of origin, with higher figures among Spaniards (64% vs. 40%, χ² (1, *n* = 90) = 5.31, *p* = 0.02). The reasons for this discrepancy warrant further investigation but may include differences in healthcare-seeking behavior and variations in provider beliefs and practices.

Our study has important limitations. Primarily, as our project largely relied on a retrospective analysis of medical records, potential inaccuracies due to incomplete or inconsistent record-keeping could affect our estimates of HIV patient characteristics. Furthermore, the absence of data specifying patient affiliation with certain key populations means we could not evaluate the potential efficacy of targeted versus opportunistic screening strategies. Instead, we could only compare indicator-condition-driven HIV diagnosis with opportunistic screening. Finally, the results may not be generalizable to regions with differing demographic characteristics or healthcare systems.

In conclusion, our findings provide valuable insights to enhance HIV testing, early diagnosis, and linkage to care. While it is essential to ensure that indicator-condition-driven HIV diagnosis is implemented as a minimum standard of practice, enhanced screening strategies are crucial to reduce late diagnoses and missed opportunities, and to effectively end the epidemic. By offering opportunistic HIV screening to all eligible patients attending healthcare services in areas with unmet need, we can help reduce stigma and improve access to prevention for vulnerable populations and asymptomatic individuals. Given the heterogeneity of our patient population, there is a clear need for integrated approaches to ensure that all individuals, regardless of age or country of origin, have equitable access to early and effective HIV care. Lastly, our results highlight the role of diverse healthcare settings in early HIV detection. Future research should explore potential barriers and facilitators to HIV testing in various clinical settings to inform quality improvement interventions.

## Data Availability

The data that support the findings of this study are available on request from the corresponding author. The data are not publicly available due to privacy or ethical restrictions.
